# Health service provider education and/or training in infant male circumcision to improve short- and long-term morbidity outcomes: protocol for systematic review

**DOI:** 10.1186/s13643-016-0216-6

**Published:** 2016-03-01

**Authors:** Thomas Gyan, Natalie Strobel, Kimberley McAuley, Caitlin Shannon, Sam Newton, Charlotte Tawiah-Agyemang, Seeba Amenga-Etego, Seth Owusu-Agyei, David Forbes, Karen Edmond

**Affiliations:** School of Paediatrics and Child Health, The University of Western Australia, Level 4, Administration Building Princess Margaret Hospital for Children, Roberts Road, Subiaco, 6008 Western Australia; EngenderHealth Inc., Greater New York Area, USA; School of Community Health, Kwame Nkrumah University of Science and Technology, Kumasi, Ghana; Kintampo Health Research Centre, Ghana Health Service, Kintampo, Ghana

**Keywords:** Health service provider, Training, Education, Infant, Male, Circumcision, Morbidity, Mortality

## Abstract

**Background:**

There has been an expansion of circumcision services in Africa as part of a long-term HIV prevention strategy. However, the effect of infant male circumcision on morbidity and mortality still remains unclear. Acute morbidities associated with circumcision include pain, bleeding, swelling, infection, tetanus or inadequate skin removal. Scale-up of circumcision services could lead to a rise in these associated morbidities that could have significant impact on health service delivery and the safety of infants.

Multidisciplinary training programmes have been developed to improve skills of health service providers, but very little is known about the effectiveness of health service provider education and/or training for infant male circumcision on short- and long-term morbidity outcomes. This review aims to evaluate the effectiveness of health service provider education and/or training for infant male circumcision on short- and long-term morbidity outcomes.

**Methods/design:**

The review will include studies comparing health service providers who have received education and/or training to improve their skills for infant male circumcision with those who have not received education and/or training. Randomised controlled trials (RCTs) and cluster RCTs will be included. The outcomes of interest are short-term morbidities of the male infant including pain, infection, tetanus, bleeding, excess skin removal, glans amputation and fistula. Long-term morbidities include urinary tract infection (UTI), HIV infection and abnormalities of urination. Databases such as MEDLINE (OVID), PsycINFO (OVID), EMBASE (OVID), CINAHL, Cochrane Library (including CENTRAL and DARE), WHO databases and reference list of papers will be searched for relevant articles. Study selection, data extraction and synthesis and risk of bias assessment using the Cochrane risk of bias assessment tool will be conducted. We will calculate the pooled estimates of the difference in means and risk ratios using random effects models. If insufficient data are available, we will present results descriptively.

**Discussion:**

This review appears to be the first to be conducted in this area. The findings will have important implications for infant male circumcision programmes and policy.

**Systematic review registration:**

PROSPERO CRD42015029345

**Electronic supplementary material:**

The online version of this article (doi:10.1186/s13643-016-0216-6) contains supplementary material, which is available to authorized users.

## Background

There has been an expansion of circumcision services in areas of Africa as part of the long-term human immunodeficiency virus (HIV) prevention strategy. This has been initiated from the results of three randomised controlled trials that showed a 60 % protective effect of circumcision during adulthood on HIV acquisition [1–4]. Despite the outcomes of these previous randomised controlled trials (RCTs), the effect of infant male circumcision on HIV risk still remains unclear. Acute morbidities associated with circumcision also include pain, bleeding, swelling, infection, tetanus or inadequate skin removal. With increased circumcision rates, there is potential for a rise in these associated morbidities that could have a substantial impact on health service delivery and patient safety.

Infant male circumcision is commonly practiced in many parts of the world, by both formal health service providers such as doctors/surgeons, medical assistants, midwives/nurses and informal health service providers including traditional circumcision providers, traditional birth attendants, religious leaders, traditional medicine men/women and other health staff. As a surgical procedure, it is expected that male circumcision be performed at the required standard by all circumcision service providers with the requisite training. For example, health service providers need to be trained on the following: how to perform circumcision in aseptic conditions, use of equipment and tools, measures to reduce morbidity, follow-up care and education to families. Yet, health service providers often do not receive the required training and education to perform these operations to an optimal standard resulting in complications ranging from 1.7 to 7.6 % [5] following infant male circumcision.

A recent prospective study of complications of neonatal circumcision in Nigeria found that complications due to male circumcision was 10.9 % and, as a result, has recommended training of health service providers to reduce the rate of complications [6]. Another study conducted in the USA among doctors performing neonatal circumcision also indicated the lack of structured training on neonatal circumcision for doctors. The study attributed the high complication rate and poor paediatric urology results to uncoordinated and informal training schemes for doctors who performed neonatal circumcisions. This study also recommended structured and formal training as strategy to reduce complications associated with infant male circumcisions [7].

It is expected that morbidities associated with infant circumcision could be reduced if health service providers are given the necessary education and/or training to perform the circumcision. Several training programmes have been developed to improve the skills of health service providers among diverse health interventions. What is not yet clear is whether the provision of education and/or training would improve health service provider skills in infant male circumcision leading to reduced rates of short- and long-term morbidity outcomes.

There has yet to be a systematic review on this topic. A recent Cochrane review in 2012 examined the effects of routine neonatal circumcision for the prevention of urinary tract infections (UTIs) in infancy. In this review, no RCT, cluster RCT or quasi-RCTs was located [8]. Another systematic review that assessed RCTs of interventions to improve the safety and efficacy of nontherapeutic male circumcision of any age was conducted in 2010, and eight RCTs were located. Only two of the studies were conducted among infants. The review did not find any studies that reported on infant circumcision and sexually transmitted infection, penile cancer, UTIs or HIV/AIDS [9].

In 2008, the World Health Organization (WHO) Department of Reproductive Health and Research funded two workshops to document infant circumcision practices in Africa [10]. One recommendation from the workshops was that further study of current infant circumcision practices was needed to inform the development of circumcision policies. As infant male circumcision services are being promoted in areas of Africa, our review will help to provide an understanding of the current literature and gaps on the effect of health service provider education and/or training for infant male circumcision on short- and long-term morbidity outcomes.

### Objectives

The primary aim of the review is to assess the effectiveness of health service provider education and/or training in infant male circumcision on morbidity or mortality outcomes in any setting.

### Review question

What is the effectiveness of health service provider education and/or training in infant male circumcision on morbidity or mortality outcomes in any setting?

## Methods/design

### Protocol development

We developed the systematic review protocol based on recommendations of the Preferred Reporting Items for Systematic Reviews and Meta-Analyses Protocols (PRIMSA-P) statement [11] and provided a populated checklist in Additional file 1. The review protocol was revised by the authors and is registered on the PROSPERO database (CRD42015029345 which is available at http://www.crd.york.ac.uk/prospero/display_record.asp?ID=CRD42015029345).

#### Types of studies

RCTs and cluster-RCTs will be included for the review.

If possible, we will include published abstracts if there is sufficient information to allow us to assess study eligibility and risk of bias. If sufficient information is not available, the study would await assessment pending the publication of the full trial report or the provision of further information by trial authors.

#### Participants

Formal and informal health service providers will be included. Formal health service providers are professionally trained, licensed and regulated providers of medical services and include the following:

- Doctor
- Surgeon
- Medical assistant
- Midwife
- Nurse.

Informal health service providers are untrained, unlicensed, unregulated or informal private providers of medical services and include the following:

- Traditional circumcision providers
- Traditional birth attendants
- Religious leaders
- Traditional medicine men/women
- Other health facility staff.

Health service providers will be excluded if they are undergoing medical training.

### Types of interventions

#### Intervention

The intervention is an additional education and/or training to improve health service provider skills in infant male circumcision. Training could be peer-to-peer, hands-on experience, lectures, practical training, demonstrations, small-group discussions, structured training, e.g. didactic learning, or simulation training. Training could occur in any environment, e.g. hospital, clinic, family homes or provider’s homes. Education could include topics on how to perform circumcision, use of equipment, how to ensure sterility, morbidity control, communication and follow-up care.

#### Control condition

There are two different types of control groups: (i) health service providers who have not received education and/or training and (ii) health service providers who have received standard education and/or training.

Studies of complex training interventions (in which training is combined with a larger health system intervention) will be excluded.

### Types of outcome measures

Our main (primary) outcomes are as follows:

- Short- and long-term all-cause morbidities which may include pain, bleeding, excess skin removal, glans amputation, fistula, infection, tetanus, urinary tract infection (UTI), HIV infection, abnormalities of urination and other identified morbidities.
- All-cause mortality.

Short-term morbidity is defined as a morbidity occurring within 6 months of the circumcision procedure. Long-term morbidity is defined as greater than 6 months from the time of the circumcision procedure.

Our secondary outcomes are defined as follows:

- Presentations to clinic or hospital
- Family satisfaction
- Health service provider satisfaction
- Skill improvement in health service provider
- Knowledge improvement in health service providers
- Training costs.

### Search strategy

The databases to be used for searching the relevant trials include the Cochrane Central Register of Controlled Trials (CENTRAL) (The Cochrane Library), MEDLINE (Ovid), EMBASE (Ovid), and Cochrane Database of Systematic Reviews and Database of Abstracts of Reviews of Effects (DARE). An example of the MEDLINE search strategy is in Additional file 2.

The clinical trial registries include the following: ClinicalTrials.gov (http://clinicaltrials.gov/), International Standard Randomised Controlled Trial Number (ISRCTN) (http://www.controlled-trials.com), WHO International Clinical Trials Registry Platform (http://who.int/ictrp/en/) and UK Clinical Research Network Study Portfolio (http://public.ukcrn.org.uk/search/).

The search period will be from 1985 to 2015 in all languages. Translation assistance will be sought.

### Searching other sources

We will hand search reference lists from relevant articles chosen for potential inclusion in this review to identify further relevant studies. We will contact authors of included studies to determine whether there are any additional studies published, ongoing or unpublished that may be relevant.

### Study selection

All review titles and abstracts retrieved through the search strategy will be reviewed independently by two authors to identify studies that meet the inclusion criteria. Inclusion criteria at the title and abstract review level will be limited to any primary study (quantitative) reporting on intervention with focus on health service provider’s education and/or training to improve provider skills on infant male circumcision. Exclusion criteria will include infants greater than 12 months, review articles, qualitative and opinion articles. Once articles have been identified, full-text articles will be retrieved and independently assessed by two independent review authors. For any disagreement, a third reviewer will be asked to assess the trial to determine eligibility for inclusion in the review. Authors will also be contacted for further clarification if necessary. We will document reasons for exclusion, and reference manager Endnote X7 will be used during this selection process.

### Data extraction and management

A data extraction form will be developed and pre-tested before it will be used to extract data from the eligible studies. Two review authors will independently extract data from the included studies. Any disagreement between the two authors will be discussed. Further disagreement will be resolved through discussion with a third reviewer. Retrieved information will be considered based on context, intervention and outcome and includes the following.

- Methodology
  - Type of randomisation
  - Study setting
  - Recruitment
  - Loss to follow-up rates
- Study population
  - Participant’s demographics
- Interventions
  - Description of the intervention and control groups
- Outcomes
  - Primary and secondary outcomes
  - Time points of measurements
  - Indicators of assessment of risk of bias
- Authors
  - Study funding
- Notes
  - Any information by the authors that may be useful

Non-English language articles will be translated. Authors will be contacted for additional information if required. All disclaimer statements will be assessed.

### Risk of bias assessment

The Cochrane risk of bias assessment tool [12] will be used to assess the risk of eligible studies by two review authors. In case of disagreement, the third author will be involved in the assessment and resolution of the risk of bias assessments. The assessment will be categorised as either low, high or unclear risk of bias with explanation for each domain. The risk of bias domains include random sequence generation (selection bias), allocation concealment (selection bias), blinding of participants and personnel (performance bias), blinding of outcome assessment (detection bias), incomplete outcome data (attrition bias), selective reporting (reporting bias) and other bias.

Authors will be contacted to provide additional information if data for assessment of risk of bias from eligible studies is inadequate. These data will be included in the data extraction processes. We will not exclude studies based on risk of bias assessment. The information will be used in the analysis and report of the review findings.

### Overall risk of bias

The risk of bias summary tool will be used to collate the risk of bias for all eligible studies. Studies with a high or unclear risk of bias in the domain for sequence generation, similarity of baseline outcome measurements, completeness of outcome data and other risks of bias will be considered high risk. Conclusions will also take into account the impact of the bias and whether it is likely to bias the findings of the study.

### Measures of treatment effect

Data will be entered into RevMan 5.3 software. Risk ratios (RR) and 95 % confidence intervals will be calculated for dichotomous data including the proportion of infants reported as having short- or long-term morbidity outcomes, infant mortality, presentations to clinic or hospital, skill improvement as a result of the intervention, and training costs.

We will report mean difference (MD) for continuous outcomes and standardised mean difference (SMD) if different scales are used (such as family satisfaction, health service provider satisfaction and increased knowledge) with 95 % confidence intervals for outcomes in each of the studies. The level of significance will be provided in case the above is not available. Chi-square test will be used to assess heterogeneity.

### Unit of analysis

It is possible that cluster RCTs may be included in this review. If they are to be included in the meta-analysis, we will first determine if the authors have appropriately controlled for effects of clustering in the study. If there is doubt, the authors will be contacted for clarification. If the error has not been corrected and the data are available, we will derive an estimate of the intracluster correlation coefficient (ICC) from the study. If the data are not available, we will determine the ICC using a similar trial or from a study with a similar population.

We will report whether an ICC has been used and conduct a sensitivity analysis to determine the effect of using an ICC. If the clustering has been accounted for, we will determine whether this has been adequately completed. Where authors have controlled for clustering, there is little difference between the study designs or where there is unlikely to be an interaction between the effect of the intervention and the choice of randomisation method, the data from cluster RCTs may then be combined with data from individual RCTs. If we are unable to adjust for incorrect statistical methods used by the cluster trials and cannot estimate the ICC with any a degree of confidence, we will exclude the trial [12].

### Dealing with missing data

We will attempt to contact authors by email for any missing data.

### Assessment of heterogeneity

We will construct forest plots to examine heterogeneity between interventions and quantify the impact of the heterogeneity using the *I*^2^ and chi-square statistics. If we identify a substantial level of heterogeneity in trials (for example, the *I*^2^ is more than 30 to 60 %, the *P* value is less than 0.10 in the chi-square test for heterogeneity or there is a different magnitude and direction of effects), we will perform subgroup analyses to explore the possible causes of statistical heterogeneity [12] .

### Assessment of reporting biases

If we are able to pool more than ten trials, we will create and examine a funnel plot to explore possible small study effects and publication biases.

### Data synthesis

Where data is available, we will perform a single meta-analysis for each outcome using RevMan 5.3 software. For each outcome, we will firstly group by the type of control and analyse each group separately. If there are no differences, we will combine the two groups for an overall analysis. For quantitative data, we will calculate the pooled estimates of the SMDs or MDs and 95 % confidence intervals using the random effects model. We will also calculate pooled RR estimates and 95 % confidence intervals using the random effects model. Where statistical pooling is not possible, the findings will be analysed descriptively.

### Assessing methodological quality

We will use the Grades of Recommendation, Assessment, Development, and Evaluation (GRADE) approach to assess the quality of the outcomes reported in this review [13].

### Subgroup analysis and heterogeneity

We will undertake subgroup analysis for the primary outcome measures if significant heterogeneity is identified in the trials for the following categories: formal and informal health service providers and low-, middle- and high-income countries. PROGRESS-Plus is an assessment of participant’s socio-demographic characteristics and subgroup outcomes (including socio-economic status, provider education, occupation of provider, ethnicity, religion) [14], settings of procedure, location of provider, risk groups of health system non-regulation or recognition, such as informal providers particularly traditional providers.

### Sensitivity analysis

We planned to conduct sensitivity analysis to assess the impact of excluding studies with a high risk of bias assessment on the primary outcomes.

## Discussion

The findings of our review will have significant implications for infant male circumcision programmes and policy in any setting. Both formal and informal health service providers will continue to play important roles in the provision of infant male circumcision services globally. The association between poverty, infant health status and access to appropriate health care is relevant for decisions regarding the education and/or training to improve health service provider skills on infant circumcision to reduce morbidity in any setting. In settings where there are few formal health service providers and/or poor access to health facilities and families continue to seek services from diverse health service providers, education and/or training may be an effective way to reduce both short- and long-term morbidity outcomes. Similarly, in places where access and high-quality health service system are available but families decide to rely on diverse health service providers for services, education and/or training and structured collaboration with the health system may be an effective way to reduce morbidity associated with infant male circumcision.

## Additional files

Additional file 1: PRISMA-P (Preferred Reporting Items for Systematic review and Meta-Analysis Protocols) 2015 checklist. (DOXC 15 kb) Additional file 2: Search strategy MEDLINE. (DOXC 83 KB)

## Abbreviations

AIDS: acquired immunodeficiency syndrome
HIV: human immunodeficiency virus
KHRC: Kintampo Health Research Centre
MD: mean difference
RCT: randomised controlled trial
RR: risk ratio
SMD: standardised mean difference
SPACH: School of Paediatrics and Child Health, University of Western Australia
UTI: urinary tract infection
WHO: World Health Organization
