# Supplementary material for: Health service provider education and/or training in infant male circumcision to improve short- and long-term morbidity outcomes: protocol for systematic review
Source: Syst Rev. 2016 Mar 1;5:41. doi: 10.1186/s13643-016-0216-6 (PMC4774100; doi:10.1186/s13643-016-0216-6)
Supplement: Additional file 2: — Search strategy MEDLINE. (DOXC 83 KB) [file 13643_2016_216_MOESM2_ESM.docx]

Additional file 2: Search Strategy MEDLINE

Language: all

Date: 1985 to 2015

Search Strategy:

| **Set**  **#** | **Search string** | **Explanation** |
| --- | --- | --- |
| 1 | ((lay or voluntary or volunteer? or untrained or unlicensed or nonprofessional? or non professional?) adj5 (worker? or visitor? or attendant? or aide or aides or support$ or person$ or helper? or carer? or caregiver? or care giver? or consultant? or assistant? or staff or visit$ or midwife or midwives)).tw. | Informal health service providers |
| 2 | Community Health Aides/ | Informal health service providers |
| 3 | barefoot doctor?.tw. | Informal health service providers |
| 4 | ((community or village?) adj3 (health worker? or health care worker? or healthcare worker?)).tw. | Informal health service providers |
| 5 | "Health Personnel"[Mesh] OR "Personnel, Hospital"[Mesh] OR "health care worker"[tw] OR "health care workers"[tw] OR "health care personnel"[tw] OR "health personnel"[tw] OR "health-personnel"[tw] OR "health provider"[tw] OR "health providers"[tw] OR "health care provider"[tw] OR "health care providers"[tw] OR "health staff"[tw] OR "health care staff"[tw] OR "healthcare staff"[tw] OR "health professional"[tw] OR "health care professional"[tw] OR "healthcare professional"[tw] OR "health worker"[tw] OR "medical staff"[tw] OR "medical personnel"[tw] OR "medical professional"[tw] OR "medical worker"[tw] OR "medical workers"[tw] OR "medical provider"[tw] OR OR "Physicians"[Mesh] OR "physician"[tw] OR "physicians"[tw] OR "doctor"[tw] OR "practitioner"[tw] OR "clinician"[tw] OR "nursing staff"[tw] OR "Nurses"[Mesh] OR "nurse"[tw] OR "nurses"[tw] OR "nursing assistant"[tw] OR "nursing assistants"[tw] OR "Nurses' Aides"[Mesh] OR "Nurse Midwives"[Mesh] OR "midwife"[tw] OR "midwives"[tw] | Formal health service providers |
| 6 | (newborn$ OR neonat$ OR neo-nat$ OR infan$ OR baby OR babies OR pediatric$ OR paediatric$) AND boy$ [tw] | Population |
| 7 | education*[tw] OR train*[tw] OR learn*[tw] OR teach*[tw] OR course*[tw] | Intervention |
| 8 | randomized controlled trial [pt] OR controlled clinical trial [pt] OR randomized [tiab] OR placebo [tiab] OR clinical trials as topic [mesh: noexp] OR randomly [tiab] OR trial [ti] | Randomised control trial search |
| 9 | 1 AND 2 AND 3 AND 4 AND 5 AND 6 AND 7 AND 8 |  |
